# Development of Pectin-Type B Gelatin Polyelectrolyte Complex for Curcumin Delivery in Anticancer Therapy

**DOI:** 10.3390/ijms19113625

**Published:** 2018-11-17

**Authors:** Fu-Ying Shih, Ih-Jen Su, Li-Lun Chu, Xiaojie Lin, Sheng-Chu Kuo, Yu-Chi Hou, Yi-Ting Chiang

**Affiliations:** 1 Ph. D. Program for Biotech Pharmaceutical Industry, School of Pharmacy, China Medical University, Taichung 404, Taiwan; u106308001@cmu.edu.tw; 2 Department of Biotechnology, Southern Taiwan University of Science and Technology, Tainan 710, Taiwan; suihjen0704@stust.edu.tw; 3 Department of Pathology, National Cheng Kung University Hospital, Tainan 704, Taiwan; 4 National Institute of Infectious Diseases and Vaccinology, National Health Research Institutes, Tainan 704, Taiwan; 5 School of Pharmacy, China Medical University, Taichung 404, Taiwan; u103003505@cmu.edu.tw (L.-L.C.); houyc@mail.cmu.edu.tw (Y.-C.H.); 6 Department of Chemical Engineering, University of Washington, Seattle, WA 98195, USA; xjlin@uw.edu; 7 Chinese Medicine Research Center, China Medical University, Taichung 404, Taiwan; sckuo@mail.cmu.edu.tw; 8 Research Center for Chinese Herbal Medicine, China Medical University, Taichung 404, Taiwan

**Keywords:** polyelectrolyte complex, curcumin, anticancer therapy

## Abstract

Curcumin has been proven to be a potent agent in colon cancer treatment. However, its hydrophobicity and low oral bioavailability hampered its clinical application. These limitations could be improved through appropriate formulations such as using polyelectrolyte complexes (PECs). PECs were self-assembled with polycations and polyanions in polar solvents. In this study, a novel pectin-type B gelatin PEC was developed for use in curcumin formulation. At pH 4.0, natural polyanions pectin and polycations type B gelatin spontaneously formed PECs in ethanol/water solution, whereas under mimetic gastrointestinal tract (GI tract) conditions, at pH 2.0 and 8.0, pectin and type B gelatin were electrically neutralized, and the PECs swelled to allow payload release. After being transferred to pH 7.0 condition, as in the colon environment, PECs were internalized into colon carcinomas. Thus, pectin-type B gelatin PECs were successfully prepared, and their constituent ratio and drug-loading process were also optimized. The optimum particle size of the PECs was 264.0 ± 3.1 nm and they could swell as the zeta potential was altered at either pH 2.0 or 8.0. The optimum drug content and loading efficiency were 40% and 53%, respectively. At pH 2.0, curcumin was rapidly released from curcumin-loaded PECs, whereas at pH 8.0, curcumin-loaded PECs showed a sustained-release of curcumin. The bare PECs showed very low toxicity toward human normal cells, whereas curcumin-loaded PECs, after incubation at pH 2.0 for 2 h and at pH 8.0 for 4 h, induced cell cycle arrest and exhibited cytotoxic effect to HCT116 human colon cancer cells, even though these loaded PECs were pretreated with mimetic GI tract conditions. Our pectin-type B gelatin PECs were shown to be a promising oral formulation for curcumin delivery in anticancer therapy.

## 1. Introduction

Polyelectrolyte complexes (PECs) are made from two or more macromolecules carrying opposite electrical charges, which can be ionized and electrically complexed with each other in a polar solution [1]. The polymeric acids (polyanions) or bases (polycations) respond to environmental pH value for their ionization in the pKa interval [2]. Therefore, in the field of pharmaceutics, PECs have been developed for decades to encapsulate drugs in oral-controlled or sustained-release system, in response to the environmental pH of the gastrointestinal tract (GI tract) [3]. Besides, PECs are usually formed in polar solvents, such as water or ethanol, to improve the solubility of hydrophobic drugs in pharmaceutics [4]. Among all developed polymeric materials, natural polymers attract much attention for application in drug delivery system because of their excellent biocompatibility and biodegradability [5]. The commonly used polyanions are polysaccharides, whose side chains are appended with carboxylic acids, such as pectin, alginates, and acacia gum [6]. Proteins, such as zein and gelatin, and modified polysaccharides, such as chitosan, are widely utilized as natural polycationic materials [7,8,9]. In addition, natural polymers as PECs have been applied in drug delivery systems to improve the water solubility or for controlled release designs in many studies [1]. For example, D. Wurster et al. have prepared natural polymers containing cationic type A gelatin and anionic acacia gum to generate PECs for encapsulating hydrophobic agent vitamin A palmitate. The mixing ratios and fabricating methods have been studied and optimized. The PECs could efficiently improve the water solubility of vitamin A palmitate [10]. B. Stokke et al. have built and optimized PECs with natural polymers containing cationic chitosan (pKa 6.5–6.6) and anionic alginates (pKa 3.4–3.7) at the pKa intervals, and they measured the average diameters upon environmental pH changes from pH 3.0 to 8.0. Moreover, they have found that particle sizes would significantly change at above pH 7.0 for the zeta potential alteration (above the pKa of chitosan), that would be potential in controlled releasing pharmaceutical designs [8].

Curcumin (diferuloylmethane) is a phenolic component extracted from the rhizome of turmeric [11]. As a bioactive compound, it has proven its medical efficacy in several tumors [12,13,14], including gastric [15] and colorectal cancers [16,17]. However, its low water solubility and bioavailability limit its clinical uses via oral administration [18,19]. Besides, curcumin in a buffer decomposes in a pH-dependent manner, and this degradation accelerates at neutral-basic conditions in preservation, strongly diminishing its pharmacological activity [20]. Studies have suggested that curcumin could be well formulated to improve its solubility and bioavailability via a reliable controlled- or sustained-release system [21]. PEC is one of several promising approaches and several attempts to generate PECs for curcumin delivery have been conducted [22,23]. K. Hu et al. have fabricated nano-sized zein-pectin PEC for curcumin delivery [7]. P. Sarika et al. have prepared a cationized gelatin and sodium alginate PEC to improve the water solubility of curcumin, allowing sustained-release of curcumin and enhancing its cytotoxicity in vitro [24]. A. Anitha et al. have prepared curcumin-loaded dextran sulfate-chitosan PECs to increase the water solubility of curcumin, and the curcumin content also exhibited cytotoxicity toward cancer cells [25]. Although the principle of PEC formation and studies have proved that PECs would be an applicable formulation for curcumin, it is still difficult to optimize their manufacturing process, including determination of constituent ratios and pH for aggregation [26]. These manufacturing factors strongly affect the particle size, morphology, loading efficiency, and drug-releasing behaviors of PEC systems [27]. Thus, it is essential to develop a PEC system with an optimized manufacturing process.

**Scheme 1:**
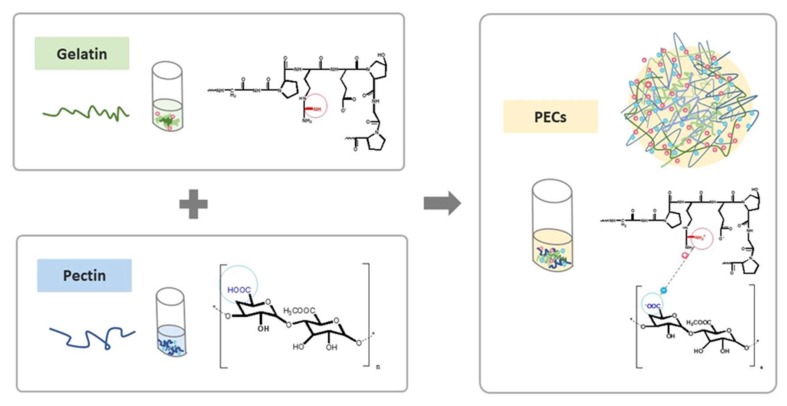
Preparation of polyelectrolyte complexes (PECs). Pectin and type B gelatin were first prepared into diluted polymer solutions in ethanol/water (1:10, *v*/*v*). At pH 4.0, pectin and type B gelatin were mixed together, spontaneously constituting PECs by their charged side chains.

In this study, we proposed a novel PEC composed of natural polymers, and its fabrication was optimized based on its performance in mimetic GI environment. We assembled the natural polycation type B gelatin and the polyanion pectin into a PEC system at an appropriate pH in a polar solvent system, as shown in Scheme 1. Several studies have previously reported pectin-gelatin PECs, yet type A gelatin was mostly chosen for use in their systems [26,28]. Type B gelatin, to our best knowledge, was first introduced in PEC for curcumin delivery. Type B gelatin is derived from collagen after lime hydrolyzation, and its isoelectric point (pI) was 4.8–5.5. The cationized type B gelatin would yield below pH 4.8, such as at pH 4.0. Apart from the electrical properties of type B gelatin, the pI of type A gelatin is approximately 9.0; thus, it is positively charged under this broadline [29]. Importantly, the pKa of polysaccharides pectin is 3.5 [30]. This pectin has been utilized to fabricate PEC because it could be electrically neutralized to force payload release in a stomach environment, which has a pH of 2.0 [31]. Besides, recent studies have pointed out the potential of pectin in colon cancer treatment because its backbone could only be cleaved by colon microorganisms [32]. Herein, PECs composed of pectin and type B gelatin were prepared at pH 4.0, which could reduce the decomposition of curcumin during manufacture or preservation. The curcumin-releasing profiles of the PECs were further investigated in the mimetic GI environmental pH value and transit periods (pH 2.0 for 2 h to simulate conditions in the stomach, and pH 8.0 for 4 h to mimic pH conditions in the small intestine) [33]. The remaining curcumin-loaded PECs after pretreatment with mimetic GI tract conditions would be internalized into the colon carcinoma. The biosafety and cytotoxic effect of curcumin-loaded PECs to human normal cells and colon cancer were examined in this study to evaluate the potentials of PECs as a curcumin formulation for future clinical anticancer treatment.

## 2. Results

### 2.1. Preparation and Characterization of Pectin-Type B Gelatin PECs

PECs were spontaneously assembled in the vicinity of the pKa or pI intervals of the two constituting polymers. The pI of type B gelatin was 4.8–5.5, indicating that the amine groups appended in type B gelatin would be electrically cationic below pH 4.8, whereas pectin, whose pKa is 3.5, would be anionized at above pH 3.5 owing to the ionization of the carboxylic groups appended on its side chains [30]. To fabricate PECs in this study, we first prepared type B gelatin and pectin into 0.1% and 0.02% *w*/*w* diluted aqueous solutions, respectively, to prevent rapid aggregation and formation of large particles. Next, we adjusted the pH value into 4.0 to allow simultaneous cationization of type B gelatin and anionization of pectin. Opposing electrical charges would spontaneously drive the type B gelatin and pectin into PECs in the polar solvent. Considering the subsequent curcumin-loading process, in this study we used ethanol (10% *v*/*v*) as a polar solvent in our PEC system. Lastly, the cationic type B gelatin, anionic pectin, and ethanol would be adjusted to optimize our PEC system.

To optimize the PEC system, the components (mainly pectin and type B gelatin) were assembled various ratios into the PECs, and particle size and distribution were measured by dynamic laser scattering (DLS). The results are presented in Table 1. Table 1 indicated that particle size significantly decreased along with reduction in type B gelatin content until only 60% of type B gelatin remained the PECs. For example, the particle size of the P1G9 sample (10% mole of pectin and 90% of type B gelatin) was 2745.0 ± 12.8 nm, while that of the P4G6 sample (40% mole of pectin and 60% of type B gelatin) decreased to 237.0 ± 3.3 nm. Because the type B gelatin content decreased by less than 60%, the particle size would not be dramatically altered. However, the polydispersity indexes (PDIs), which represents size distribution, would gradually increase with the continual decrease of type B gelatin content. To further investigate the components’ impact on electrical properties and environmental pH, we chose to use P4G6 (40% mole of pectin and 60% mole of type B gelatin) and P6G4 (60% mole of pectin and 40% mole of type B gelatin), owing to their similar particle sizes and PDIs.

### 2.2. pH Responsiveness of the PECs

**Figure 1:**
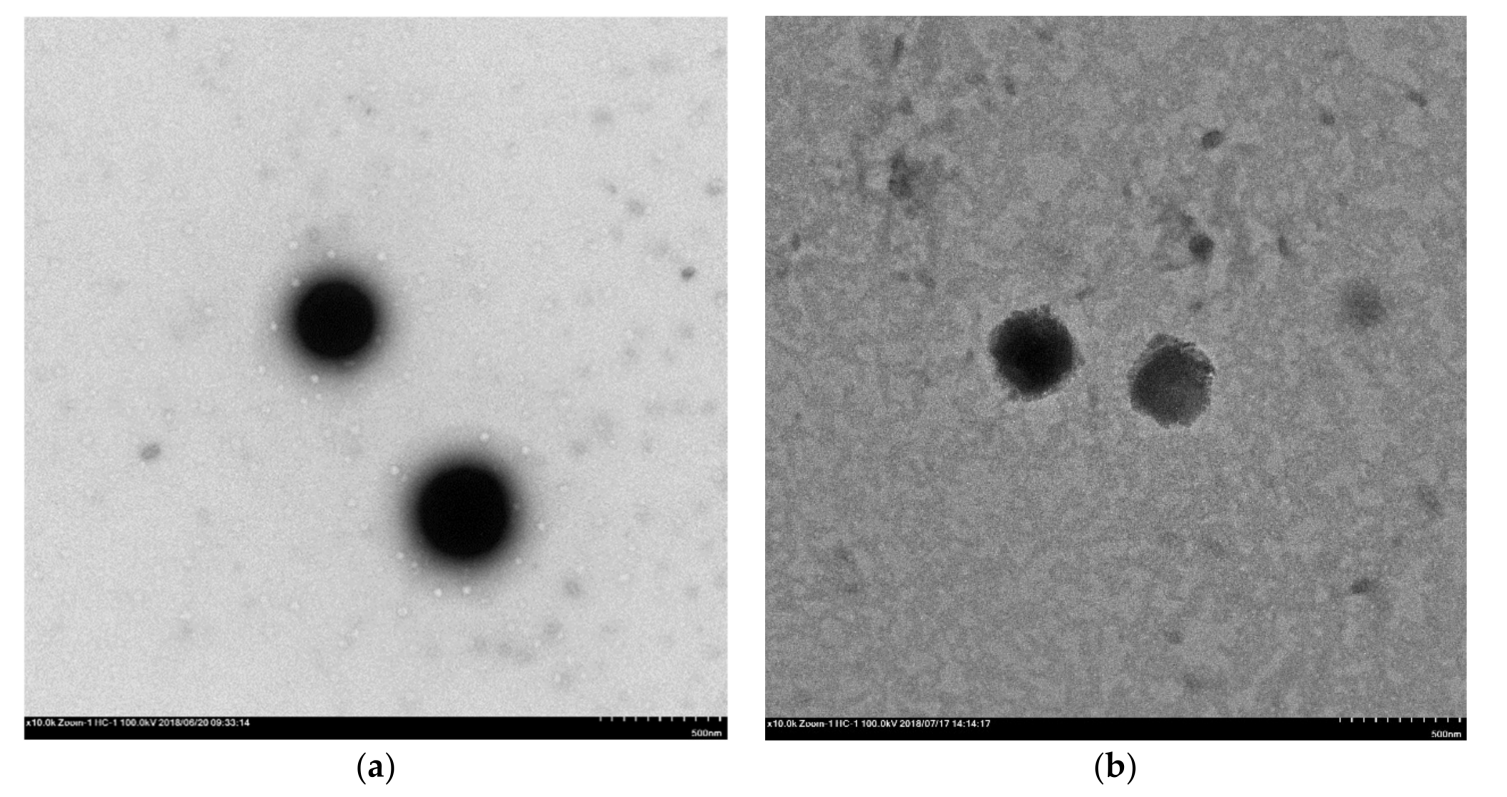
TEM images of the P4G6 (**a**) and P6G4 (**b**) PECs. The PECs were dropped to carbon-coated cooper grids. After the PECs were attached onto the carbon cooper grids, excess PECs were removed. The attached PECs were then stained with 1% sodium phosphotungstate aqueous (PTA) solution. The 1% PTA solution was dropped to the grids and excess PTA solution was removed after staining. The TEM grids were dried in a vacuum oven and stored at room temperature until observation (scale bar: 500 nm).

The particle sizes of P4G6 and P6G4 were 264.7 ± 3.1 nm and 243.1 ± 18.8 nm, respective, whereas their PDIs were both approximately 0.23. Their morphologies were observed using transmission electron microscopy (TEM) and scanning electron microscopy (SEM). The TEM images are shown in Figure 1 and they show that the observed particle sizes of P4G6 and P6G4 corresponded to their hydrodynamic diameter, as measured by DLS. However, their morphologies were different. The TEM images indicated that P4G6 particles exhibited spherical structure, whereas P6G4 particles showed irregularly spherical structure, probably as a result from the differences in their components. The morphologies of P4G6 and P6G4 PECs were also observed by a scanning electron microscopy (SEM) after the PECs were freezed dried. The SEM images of P4G6 and P6G4 were respectively present in Figure S1a,b in Supplementary Materials. P4G6 PECs exhibited dense nanosperical structure, while P6G4 PECs was getting loose after being freeze-dried. That further confirmed the components of PECs might affect the PEC structure. Both TEM and SEM images suggested that P4G6 PECs exist the stronger electrical interactions to maintain their structure, even after being freeze-dried. However, both P4G6 and P6G4 PECS could also be observed deformation by the SEM after being freeze-dried, that also suggested our PEC system should be phramaceutically delivered in a solution form. Herein, the following experiments would be perfomed and evaluated as PEC is delivered in the solution form.

**Figure 2:**
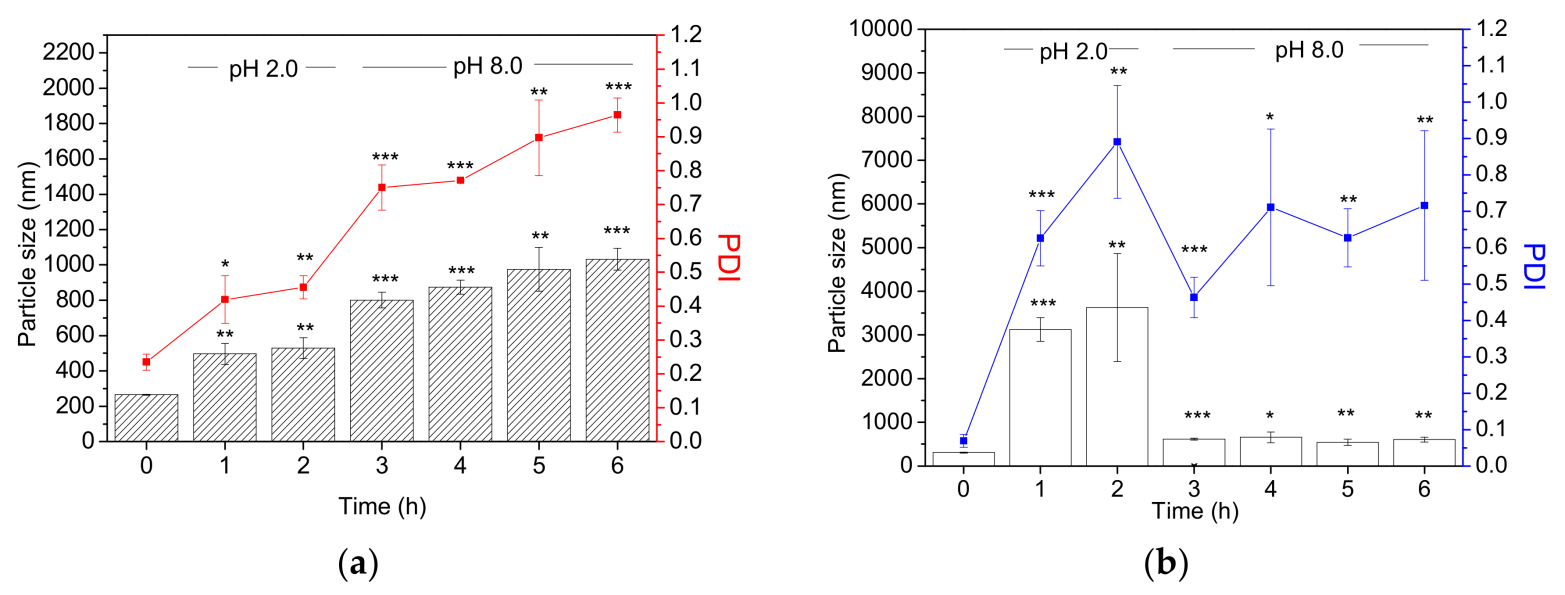
Particle size and distribution of the P4G6 (**a**) and P6G4 (**b**) PECs. The PECs were incubated at 37 °C and pH 2.0 for 2 h, and the pH was subsequently adjusted to 8.0 for 4 h to mimic drug transits in the GI tract. At each hour, particle size and PDI were determined by DLS. The results have been statistically compared and analyzed using Student’s *t*-test (Microsoft Excel 2000). Differences were recognized as statistically significant when the *p* values were less than 0.05, and significant differences are shown in star marks (* *p* < 0.05; ** *p* < 0.01, and *** *p* < 0.001).

By analyzing the TEM images, we identified that the components affected the particles’ particular shapes. Responsiveness to environmental pH would be influenced by the PEC components. In this study, we mimicked pH changes in the digesting process of drugs following oral administration. In the stomach, the pH is 2.0 on average and maintained for 2 h; in the small intestine, the pH increases to 8.0, which is retained for 4 h [33]. Herein, we simulated the pH values and periods of drug or food digestion in the GI tract following oral administration to investigate the pH responsiveness of PECs with respect to particle size, and then optimized the PEC components. The PECs were first incubated at pH 2.0 for 2 h; afterward, the PECs were incubated at pH 8.0 for 4 h. The resulting particle size and distribution are presented in Figure 2. Figure 2a,b indicated the particle size and distribution of P4G6 and P6G4 PECs, respectively, at pH 2.0 and 8.0. The trends in size and PDI alterations were marked. The P4G6 PECs showed a steady increase in particle size and PDI values at either pH 2.0 or 8.0. P6G4 PECs exhibited an abrupt increase in size and PDI at pH 2.0, although these PECs also significantly changed their hydrodynamic diameter at pH 8.0. The different performances of particle alteration could be attributed to the different ratios of pectin and type B gelatin within the PECs, resulting in different electrical properties in various pH conditions. 

**Figure 3:**
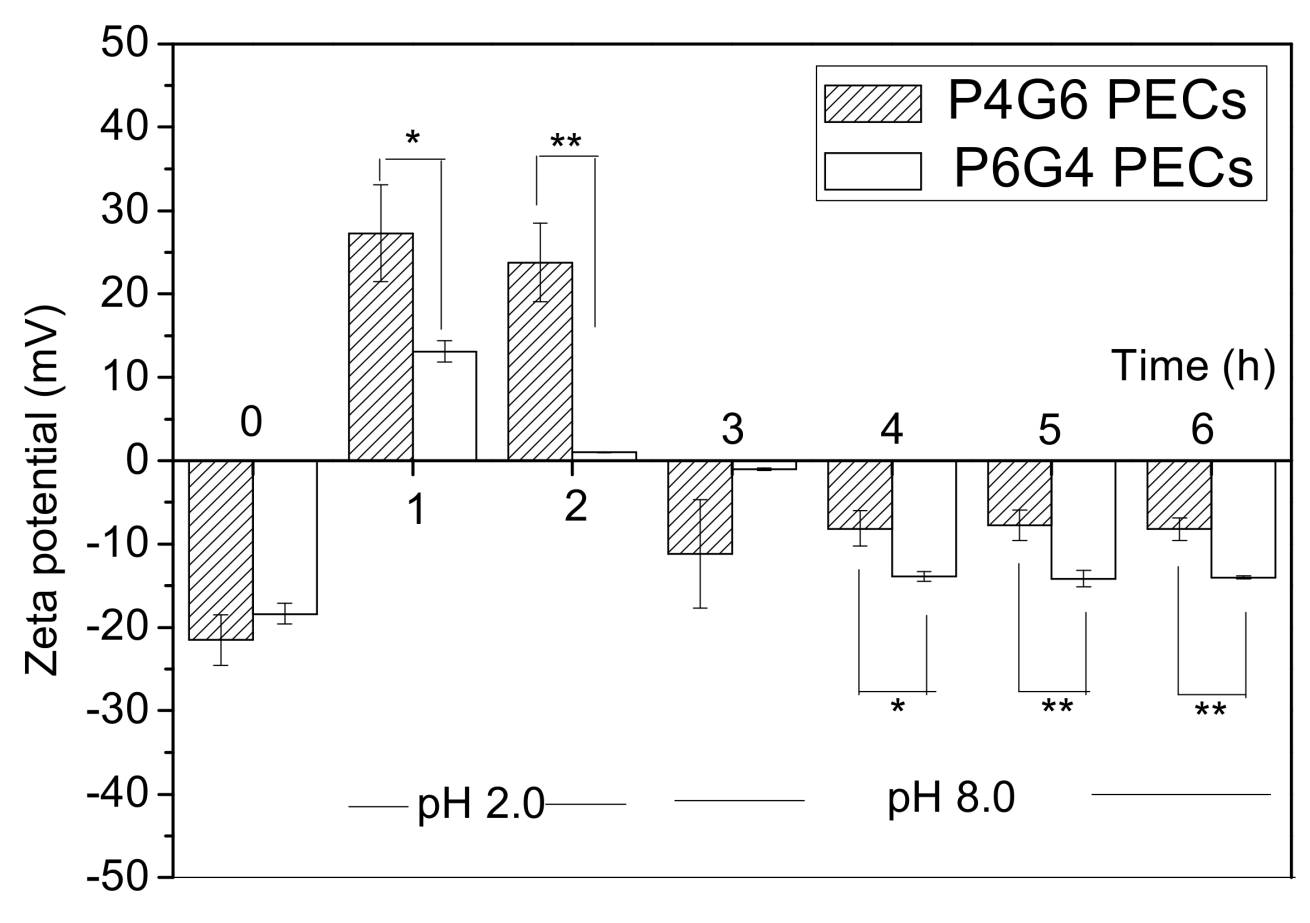
Zeta potentials of the PECs. P4G6 and P6G4 PECs were placed at 37 °C and pH 2.0 for 2 h, and the environmental pH was adjusted to 8.0 for 4 h to simulate drug transportation in the GI tract. At the predetermined time points (each hour), zeta potential was determined by electrophoretic light scattering (ELS). The results have been statistically analyzed using Student’s *t*-test (Microsoft Excel 2000). Differences were recognized as statistically significant when the *p* values were less than 0.05, and significant differences are shown in star marks (* *p* < 0.05 and ** *p* < 0.01).

Figure 3 shows the zeta potentials of P4G6 and P6G4 PECs in different pH environments, which mimicked those in the GI tract. At pH 2.0, P4G6 PECs possessed higher zeta potential than that of P6G4 PECs, whereas P6G4 PECs were more negatively charged than P4G6 PECs at pH 8.0. That may be attributed to the different ratios in the PEC compositions. At pH 2.0, pectin was neutralized owing to the protonation of the appended carboxylic groups of pectin, whereas the type B gelatin still retained its cations [29]. P4G6 PECs, which consisted of more type B gelatin, would be more positively charged. In PECs at pH 8.0, pectin was a polyanion, whereas type B gelatin would be electrically neutralized. P4G6 PECs, which comprised of less pectin, would lead to less-negative charges. P4G6 PECs could respond to the outer pH environment via superficial electrical potentials, which would not result in destruction of PECs in either acid or alkaline condition. Herein, P4G6 would potentiate a drug delivery system with the sustained-release formulation of curcumin for colon cancer treatment. 

### 2.3. Preparation and Optimization of Curcumin-Loaded PECs

**Figure 4:**
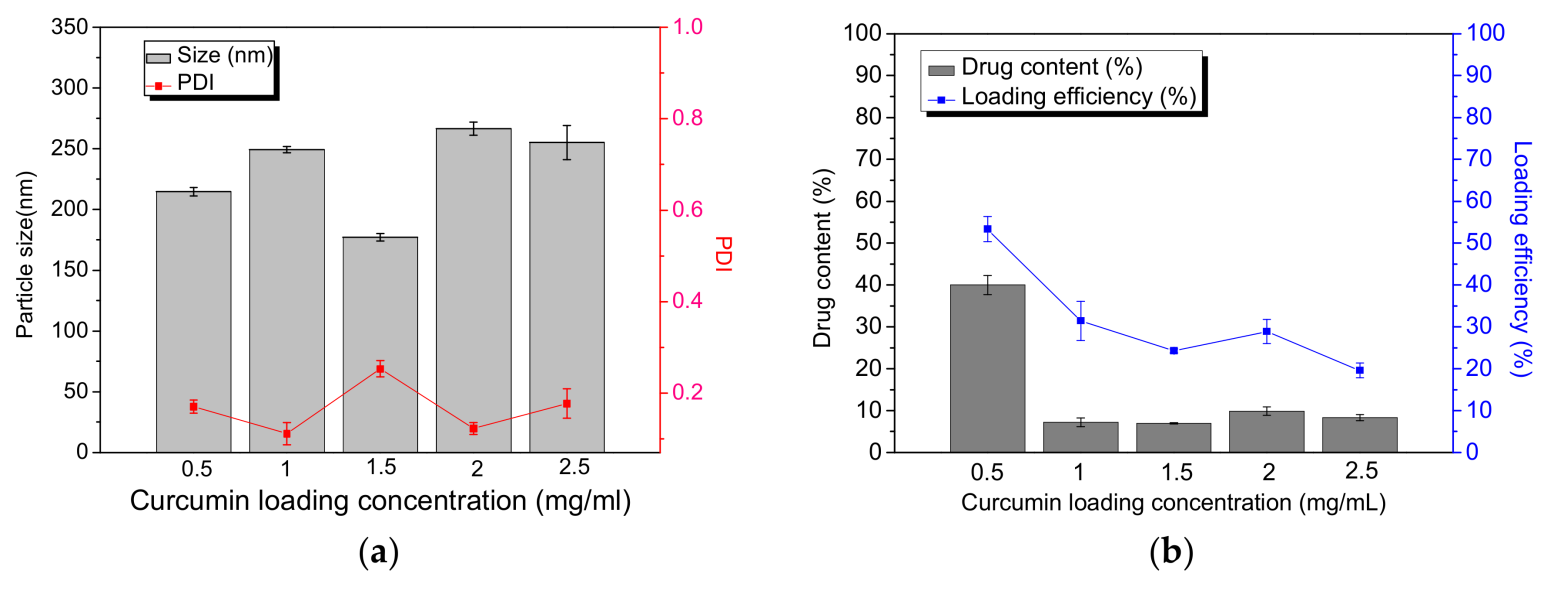
Drug-loading of curcumin-loaded PECs. Curcumin was prepared at various concentrations in alcohol. The curcumin alcoholic solution was mixed with type B gelatin and pectin, forming PECs at pH 4.0. The particle size and PDIs (**a**) of curcumin-loaded PECs were determined by DLS. To further determine drug contents and loading efficiency (**b**), the curcumin-loaded PECs were subsequently freeze-dried. The powdered curcumin-loaded PECs were reconstituted with methanol. Curcumin concentration in the solutions was measured by ultraviolet-visible light spectrum at 423 nm.

On the basis of previous results, we have chosen the P4G6 PECs as the optimum formulation. This formulation was subsequently loaded with curcumin and the loading process was also optimized. To load the hydrophobic curcumin, it was first prepared at various concentrations in ethanol, which involved our formulation as the solvent, forming 0.5, 1, 1.5, 2, and 2.5 mg/mL alcoholic solution. The alcoholic solutions were independently mixed with type B gelatin and pectin, following the abovementioned procedures. After encapsulation, the particle size and distribution of the PECs were assessed using DLS, as shown in Figure 4a. In all curcumin-loaded PECs, the particle sizes were less than 270 nm. The PECs made from 1.5 mg/mL of curcumin-loaded alcoholic solution exhibited the smallest particle size, with PDI values of 0.28, which was the highest of all PECs’. Along with the concentration of the loaded curcumin (2 and 2.5 mg/mL), particle size increased; as loading-curcumin concentration decreased (0.5 and 1 mg/mL), particle size and PDI value decreased. By ANOVA, we observed that the curcumin-loaded PECs made from 0.5 mg/mL of curcumin alcoholic solution exhibited the ideal particle size (214.67 ± 3.43 nm) and the PDI value (0.17 ± 0.01), which indicated smaller and more homogeneous particles than those of PECs without curcumin load.

Besides the particle size and PDI of the curcumin-loaded PECs, drug content and loading efficiency should also be considered to optimize curcumin encapsulation. The curcumin-loaded PECs were first freeze-dried. The entrapped curcumin was extracted using methanol from the freeze-dried powder and measured by an enzyme-linked immunosorbent assay (ELISA) reader. Drug content could be defined as the ratio of entrapped curcumin in all curcumin-loaded PECs, and loading efficiency could be determined by the ratio of loaded curcumin in comparison with the total input curcumin during the loading process. Drug content and loading efficiency are presented in Figure 4b. The highest drug content (40%) and loading efficiency (53%) were both observed in PECs made from 0.5 mg/mL of curcumin alcoholic solution. The PECs made from other loading concentrations of curcumin alcoholic solution contained less than 10% of curcumin, with loading efficiencies of lower than 35%, which resulted in a higher concentration of curcumin and interference of PEC formation [34]. The PEC made from 0.5 mg/mL of curcumin alcoholic concentration could result in not only small and homogeneous particles but also the highest drug content and loading efficiency. Herein, drug-releasing behaviors were further studied using the curcumin-loaded PECs comprising of 40% mole of pectin and 60% mole of type B gelatin, and loaded with 0.5 mg/mL of curcumin alcoholic solution.

### 2.4. Drug-Releasing Profiles

**Figure 5:**
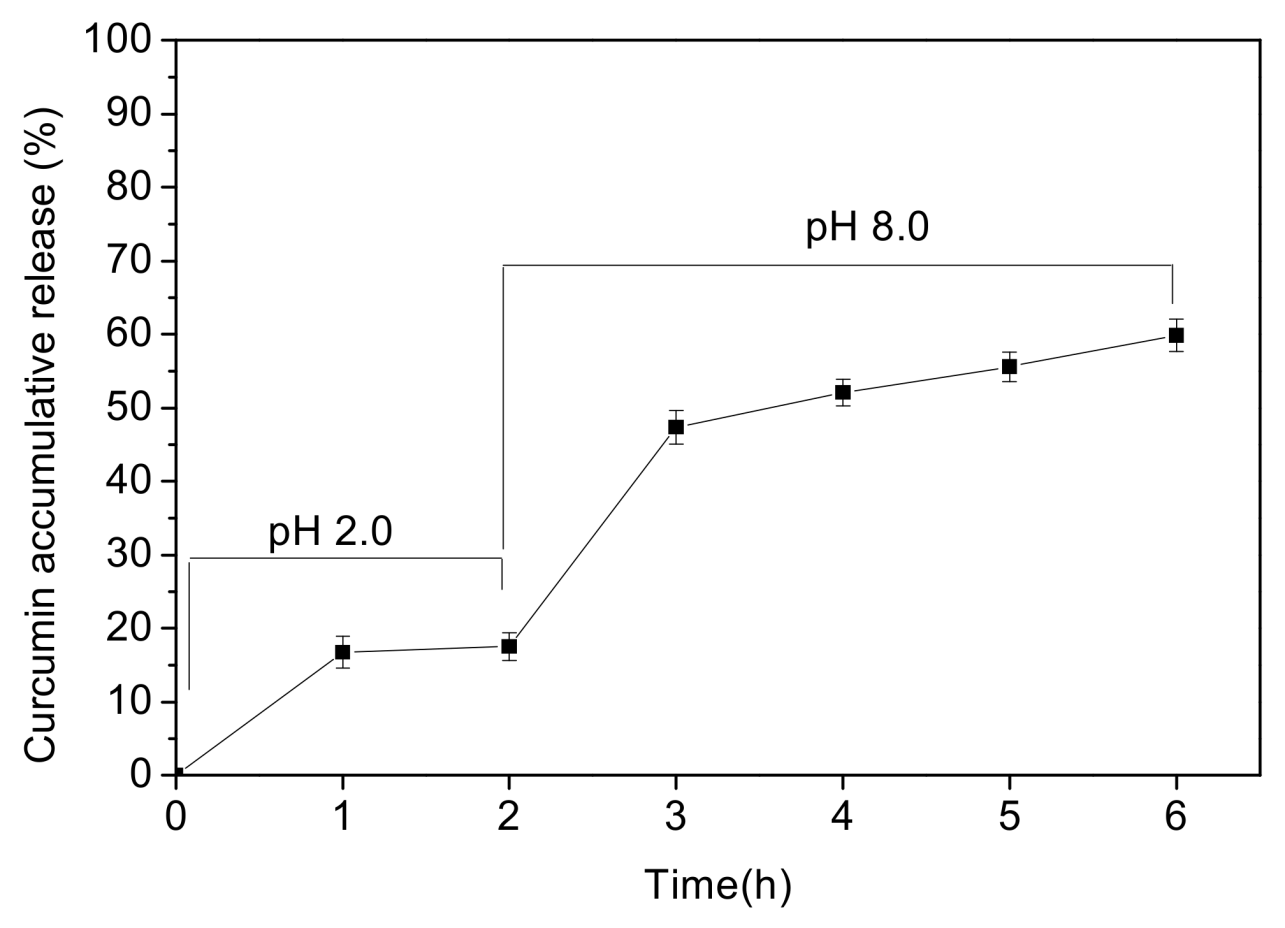
Drug-releasing profiles. Curcumin-loaded PECs were placed into dialysis bags (molecular weight cutoff: 6–8 k) (M.W.C.O.: 6–8 k), which were immersed to an ethanol/deionized water (1:1, *v*/*v*) solution at 37 °C. The pH of the outer medium was first adjusted to 2.0 for 2 h and then adjusted to 8.0 for 4 h to mimic drug transit in the GI tract. At the predetermined time points, curcumin release was determined by UV spectrum at a wavelength of 423 nm.

The drug-releasing behaviors of curcumin-loaded PECs in mimetic GI tract conditions (pH 2.0 for 2 h and pH 8.0 for 4 h) were also studied. The drug-releasing profile is shown in Figure 5, where two releasing trends are observed based on the pH values. For the first 2 h at pH 2.0, less than 20% of curcumin was released. As the pH increased to 8.0, the PECs released curcumin sustainably and eventually a total of 60% of curcumin was released. It is worth noting that the drug-releasing curve at pH 2.0 was different from that at pH 8.0. At pH 2.0, the drug-releasing curve was close to first- order drug-releasing kinetics, whereas at pH 8.0, the drug-releasing curve was close to zero-order kinetic drug-releasing profile, releasing curcumin sustainably. Besides, almost 40% of curcumin still remained inside the PECs after 6 h of acid or alkaline treatment. The residual curcumin would be provided to treat colon cancer.

### 2.5. Confocal Laser Scanning Microscopic (CLSM) Observation

**Figure 6:**
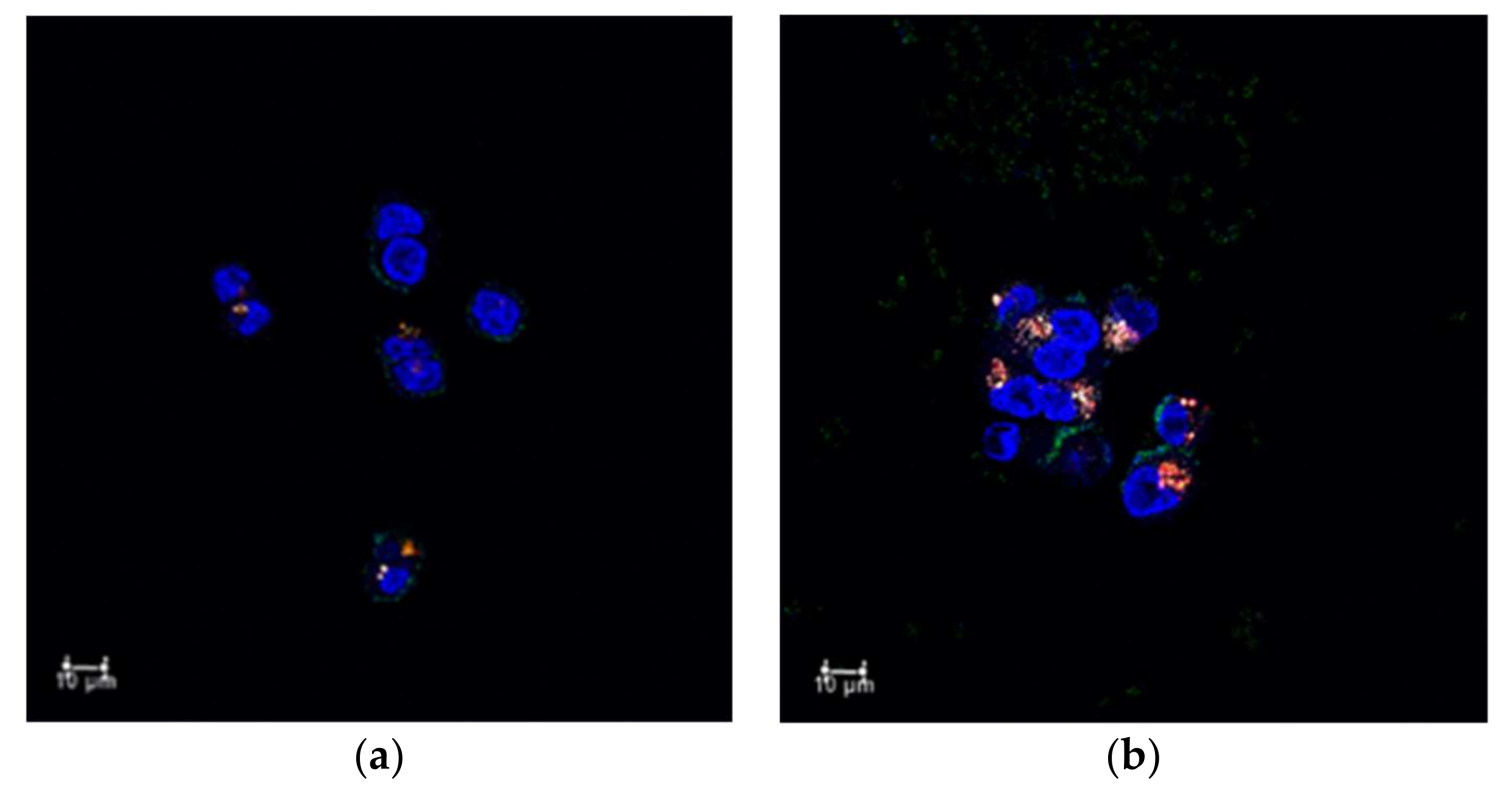
Confocal laser scanning microscope (CLSM) images showing internalization of curcumin (**a**) and curcumin-loaded PECs (**b**). HCT116 human colon cancer cells were incubated with curcumin or Cy5.5-labled curcumin-loaded PECs, which were pretreated with mimetic GI tract conditions. After 12 h of incubation, the cells were stained with Lysotracker DND-Red to label the endosome/lysosomes, and then fixed with paraformaldehyde. The fixed cells were then washed twice with PBS and further stained and mounted with 4′,6-diamidino-2-phenylindole (DAPI)-containing mounting medium. The fluorescence of the Cy 5.5-labeled curcumin-loaded PECs, curcumin, endosome/lysosomes, and DAPI was observed using a CLSM with appropriate excitation and emission wavelengths.

According to the results of drug releasing profiles (Figure 5), 60% of curcumin would be released out of the PECs in mimetic GI tract conditions. That meant 40% of the curcumin would still remain within the PECs, which has possibility to be transported to the colon lesions and uptaken by the colon cancerous cells. To confirm that the curcumin-loaded PECs could preserve curcumin and be internalized into HCT116 human colon cancer cells even after acidic or alkaline treatments, we used a CLSM for direct fluorescent observation. In order to observe the internalization of PECs to HCT116 cells, the PECs were labeled with cyanine 5.5 monosuccinimidyl ester (Cy 5.5-NHS ester) fluorescence via amide bonds with the amine groups appended on type B gelatin. The organelle endosome/lysosome was marked with Lysotracker DND-Red and cell nuclei were stained with 4’,6-diamidino-2-phenylindole (DAPI). Curcumin was detected by its own fluorescence. Curcumin and Cy 5.5-labeled curcumin-loaded PECs were incubated in mimetic GI tract conditions and added to HCT116 cells, which were then incubated for 12 h. The fluorescence of Cy 5.5, lysotracker, curcumin, and DAPI were independently excited and detected with appropriate excitation/emission wavelengths; they are shown in grey, red, green, and blue, respectively, in Figure 6. 

Figure 6 shows the internalization process of curcumin and curcumin-loaded PECs. Curcumin could enter the cells via diffusion and spread over the organelles within the cells. Herein, in Figure 6, the fluorescence of curcumin could be observed over the cell cytoplasm, even also be observed to overlap with that of endosome/lysosomes. Curcumin-loaded PECs were observed to be internalized to HCT116 cells, and mainly accumulated in the endosome/lysosomes. Curcumin fluorescence was also distributed within the cells, showing the intracellular drug-releasing behaviors of curcumin-loaded PECs. It is worth noting that the fluorescence of the free curcumin was weaker than that of curcumin-loaded PECs, leading probably from the chemical structure degradation of free curcumin. That would be an evidence for the protective effects of our PECs. The PECs would carry active curcumin into the colon cancer cells even through the mimetic GI tract conditions.

### 2.6. Cell Viability

In order to verify the therapeutic efficacy of curcumin-loaded PECs against colon cancer cells, HCT116 cells were incubated with curcumin-loaded PECs or free curcumin. The free insoluble curcumin was first prepared in 1% of dimethyl sulfoxide (DMSO), to the same concentration as curcumin concentration in the curcumin-loaded with PECs’. Both curcumin-loaded PECs and free curcumin were pretreated with mimetic GI tract conditions and transit periods, as mentioned previously. After that, curcumin and curcumin-loaded PECs were prepared at concentrations of 45 to 1.41 µM by serial dilution and respectively added to HCT116 cells. The resulting cell viability was determined by (3-(4,5-dimethylthiazol-2-yl)-2,5-diphenyltetrazolium bromide) (MTT) assay after incubation. 

**Figure 7:**
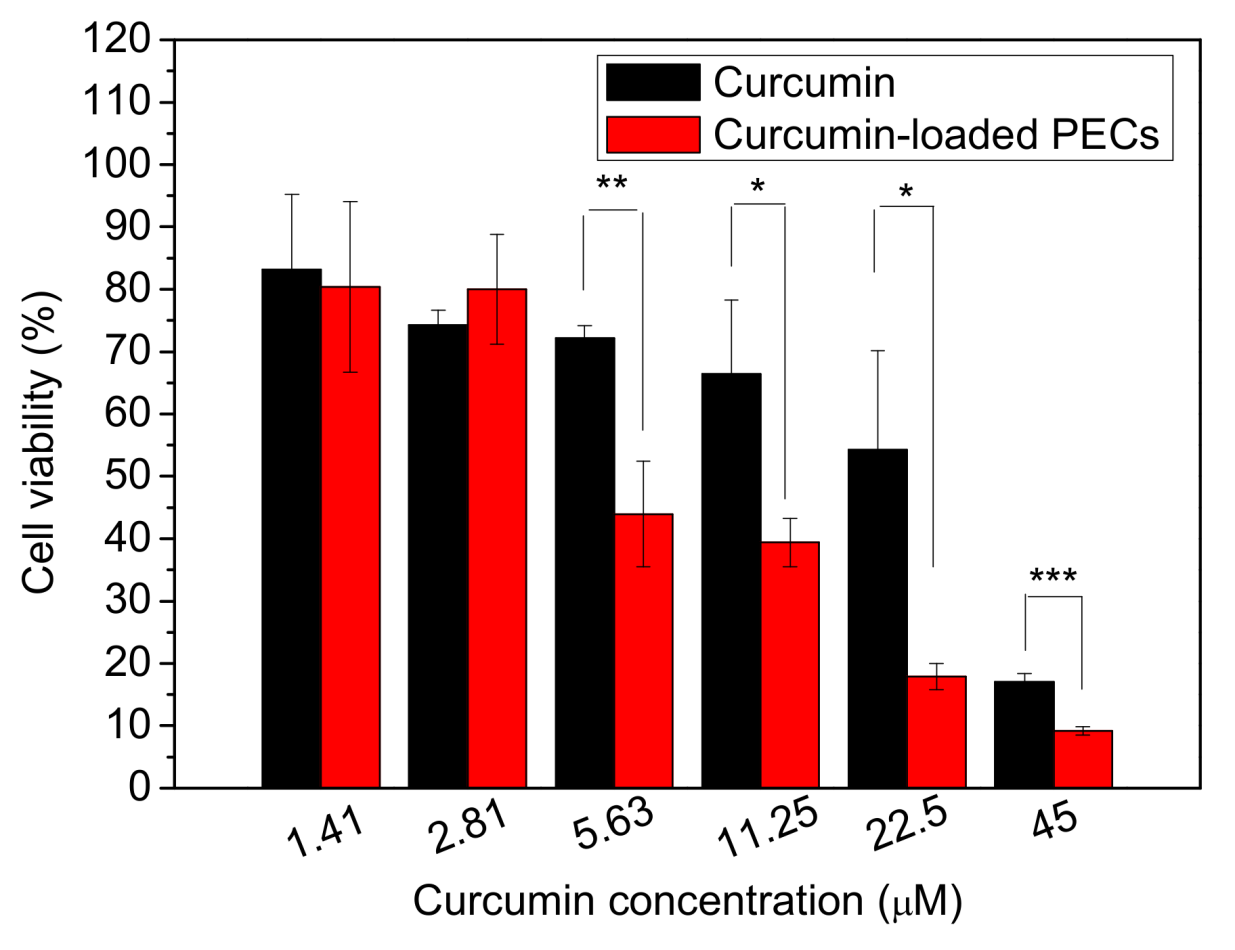
Cell viability. Curcumin and curcumin-loaded PECs were pretreated with pH 2.0 for 2 h and pH 8.0 for 4 h. Afterward, the pH values were adjusted back to 7.4. Next, HCT116 human colon cancer cells were incubated with the curcumin and curcumin-loaded PECs at 37 °C with 5% CO_2_ supply for 12 h. The curcumin and curcumin-loaded PECs were removed and the MTT assay was utilized to determine cell viability. The results have been statistically compared and analyzed using Student’s *t*-test (Microsoft Excel 2000). Differences were considered to be statistically significant when the *p* values were less than 0.05, and significant differences are shown in star marks (* *p* < 0.05; ** *p* < 0.01, and *** *p* < 0.001).

The viability of HCT116 cells following treatment with curcumin and curcumin-loaded PECs, which were pretreated with mimetic GI tract conditions, is shown in Figure 7. The results indicated that both curcumin and curcumin-loaded PECs exhibited concentration-dependent toxicity to HCT116 cells. The half maximal inhibitory concentrations (IC_50_) of free curcumin and curcumin-loaded PECs were approximately 25 and 5 µM, respectively. The results directly showed that curcumin would be degraded in mimetic GI tract conditions, thereby reducing its toxicity to colon cancer cells. Curcumin-loaded PECs enabled sustained-release of their payloads in mimetic GI tract conditions, and the released payloads exhibited toxicity to cancer cells. Besides, the PECs protected curcumin from degradation due to environmental pH during treatment with mimetic GI tract conditions; hence, more bioactive ingredients were able to arrive at tumorous lesions in the colon. Herein, the curcumin-loaded PECs exhibited higher toxicity toward HCT116 human colon cancer cells than free curcumin. 

Besides, the safety of the novel bare PECs was also evaluated using Detroit 551 human normal fibroblast cells, and the MTT assay was employed to determine cell viability. The concentrations of PECs were adjusted on the basis of drug contents until they were equal to those used for curcumin encapsulation. Cell viability data are presented in Figure S2 in the Supplementary Materials. Bare PECs exhibited very low toxicity toward human normal fibroblasts after they were directly cultured with the cells. Even though the bare PECs were incubated after treatment with mimetic GI tract conditions, as described above, the bare PECs exhibited very low cytotoxicity. The results clearly illustrated that our pectin-type B gelatin PECs possessed high safety to human normal cells, and that the bare PECs were digested after acid or alkaline treatments. 

### 2.7. Cell Cycle Analysis

**Figure 8:**
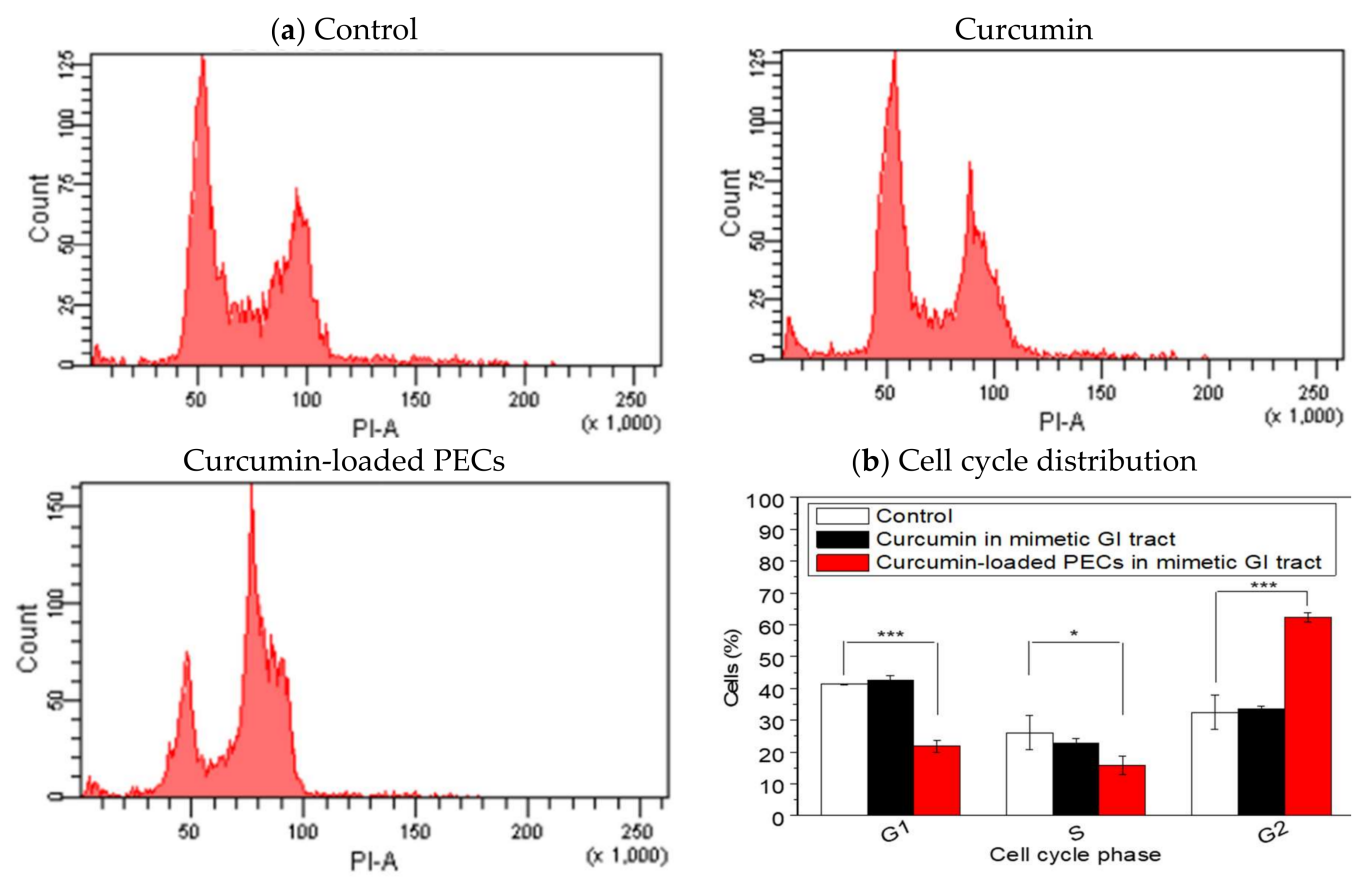
Cell cycle profiles of HCT116 cells treated with curcumin and curcumin-loaded PECs after incubation in mimetic GI tract conditions. (**a**) Curcumin and curcumin-loaded PECs (curcumin concentration was 6.8 µM) were pretreated with mimetic GI tract conditions, and HCT116 human colon cancer cells were then treated and the curcumin or curcumin-loaded PECs for 12 h. The cells were then fixed and stained with PI and cell cycle was analyzed using flow cytometry; (**b**) Cell cycle distribution was also analyzed using ModFit LT 3.0. The results have been statistically compared and analyzed using Student’s *t*-test (Microsoft Excel 2000). Differences were recognized as statistically significant when the *p* values were less than 0.05, and significant differences are shown in star marks (* *p* < 0.05 and *** *p* < 0.001).

In order to further identify the relevance of the cytotoxicity and protective ability of our PECs from exteriors, HCT116 cells were treated with curcumin or curcumin-loaded PECs, which were both preincubated in mimetic GI tract conditions (pH 2.0 for 2 h and pH 8.0 for 4 h). The cell cycle of HCT116 cells was analyzed using flow cytometry after cell fixation and staining by propidium iodide (PI). The results are shown in Figure 8. Figure 8a shows that curcumin after incubation in mimetic GI tract conditions did not cause cell cycle arrest, whereas Figure 8b indicates that curcumin-loaded PECs caused G2/M phase arrest. The results were consistent with the cytotoxic results, as shown in Figure 7 Curcumin-loaded PECs could maintain curcumin bioactivity from the environment, leading to stronger cytotoxic effects of curcumin on colon cancer cells.

## 3. Discussion

Curcumin is a potent anti-cancer compound, although its clinical application is limited owing to its hydrophobicity, poor oral bioavailability, and unstable chemical structure. To overcome these challenges, we aimed to design an appropriate curcumin oral formulation for colon cancer treatment. PECs are one of the feasible approaches for achieving this formulation. 

PEC is composed of two or more oppositely charged polymers in polar solvents. PEC could not only improve the water solubility of the encapsulated agents but also protect its payload from the environment. Besides, the electrical charge of PEC would change with the environments, thus potentiating oral-controlled or sustained-release drug delivery system using suitable constituents of PEC to enhance oral bioavailability of drugs. In this study, anionic pectin and cationic type B gelatin used to form PEC for curcumin formulation. Pectin-gelatin complexes have been developed [34]; however, there is a distinction between them and our pectin-type B gelatin PECs. First, our pectin-type B gelatin PECs were nano-sized (264.0 ± 3.1 nm), whereas the PECs reported by other research was micro-sized; hence, they are also called complex coacervates. This means that the aggregation that occurred during their manufacture would be different and new, requiring further exploitation. Besides, previous studies mostly reported type A gelatin as the polycation that interacts with polyanionic pectin. The pI of type A gelatin was 9.0, indicating that at pH 7.0, it is positively charged and capable to self-assemble with pectin (pKa 3.0) to form PECs. As pH increased over 9.0, type A gelatin is electrically neutralized. However, the highest pH in the small intestine is approximately 8.0, far lower than the pI of type A gelatin; thus, the drug-releasing site would be mostly only the upper GI tract, such as the stomach. However, D. Madhavi et al. have suggested sustained-release of curcumin via oral administration, which could increase 10-fold its bioavailability compared to that of unformulated curcumin [35]. The drug-releasing efficacy and relative bioavailability of pectin-type A gelatin PEC system would be considered. In this study, type B gelatin was first employed in the PEC system. The pI of type B gelatin was 4.8–5.5 until the pH decreased to 4.0, whereas pectin, whose pKa is 3.0, would be self-aggregated with type B gelatin. At pH 2.0, such as in the stomach, the pectin-type B gelatin complex would swell, as shown in Figure 2, because of the electrically neutralized pectin. The positively charged zeta potential is shown in Figure 3. As the pH increased to 8.0, type B gelatin was electrically neutralized and the zeta potential of the PECs was negatively charged, breaking the electrical balance of the PEC (Figure 3). Increased particle sizes and PDI were therefore observed (Figure 2). The accumulative drug-releasing profile of curcumin-loaded pectin-type B gelatin PECs also revealed their continuous drug-releasing behavior, as shown in Figure 5. Curcumin was not only abruptly released at pH 2.0 but also released sustainably at pH 8.0, which has not been commonly discussed in other studies. The releasing behavior of our PECs differed from that of pectin-type A gelatin PECs. I. Joseph et al. revealed that micro-sized pectin-type A gelatin PECs release their payloads following sustained release at pH 2.0 [36], whereas our PECs rapidly released curcumin at pH 2.0 within 1 h. This finding also evidenced the differences in drug-releasing profiles with different components of the PECs.

Micro-sized PECs (complex coacervates) could bear higher drug contents and loading efficacy after optimization, compared to our PECs. M. Saravanan et al. have prepared pectin-gelatin complex coacervation with particle size of 94 μm and high drug content and loading efficiency, which are 57% and 76%, respectively [26]. Pectin-gelatin complex coacervates were also fabricated by D. Silva el at., with drug content and loading efficiency of 56.7% and 89.5%, respectively [28]. In this study, the drug content and loading efficiency of pectin-type B gelatin PEC were also discussed. The optimum drug content and loading efficiency were 40% and 53%, respectively, whereas the particle size after encapsulation was 214.67 ± 3.43 nm with narrow particular distribution (PDI was 0.17), as presented in Figure 4. The low drug contents and loading efficiency were due to the small cavity of nano-sized PECs. The curcumin-loaded nano-sized PECs prepared by P. Sarika et al. also had low drug content and loading efficiency, compared to those of complex coacervates [24]. The particle size of our curcumin-loaded PECs was slightly smaller than that of bare PEC. The reasonable explanation would be the additional ethanol in the manufacture of these loaded PECs. The ethanol dissolved the curcumin, instead of dispersing it before loading [37]. Thus, the curcumin would not rapidly aggregate inside the core of PECs during manufacture, resulting in similar particle sizes of PEC after curcumin encapsulation.

The drug-releasing profiles of PECs in mimetic GI tract conditions were also investigated in our study, as shown in Figure 5. Curcumin-loaded PECs exhibited abrupt release of curcumin at pH 2.0 and the releasing curve was close to the first-order releasing profiles. At pH 8.0, the curcumin-loaded PECs exhibited similar curcumin releasing profiles with zero-order releasing mechanism. I. Joseph et al. have suggested that the constituents of PECs considerably influence their drug-releasing profile [36]. The drug-releasing profile of our PECs corresponded to the increase in particle size of the bare PECs containing the same ratios of components, as presented in Figure 2a. Particle sizes were observed to abruptly increase as the pH was adjusted to 2.0, which could be attributed to rapid drug-releasing profile at pH 2.0; the particle size became gradually larger at pH 8.0, indicating the sustained-release behavior of the curcumin-loaded PECs. Moreover, the curcumin-loaded PECs preserved almost 40% of payloads within the PECs, which could directly treat colon cancer cells in the GI tract, and the released curcumin would enter the blood via the GI tract to achieve anti-cancer effect. The remaining curcumin-loaded PECs after passing through the mimetic GI tract conditions have been identified that they could be internalized into the colon cancerous cells and further perform the intracellular drug releasing, shown in Figure 6.

The cytotoxicity of the curcumin-loaded PECs to HCT116 human cancer cells was accessed by MTT assay, as presented in Figure 7. In this study, we utilized 1% of DMSO to prepare curcumin solution, which could eliminate the cytotoxicity of DMSO itself [37]. The cell viabilities shown in Figure 7 have indicated that curcumin-loaded PECs exhibited stronger toxicity to HCT116 human colon cancer cells than free curcumin after treatment with mimetic GI tract conditions. The cytotoxicity might be attributed to the anti-cancer activity of curcumin. Y. Wang et al. have shown that active curcumin is degraded in a pH-dependent manner; thus, the alkaline (pH 8.0) treatment might reduce the anti-cancer activity of curcumin [20]. Curcumin-loaded PECs allowed sustained release of curcumin during digestion to maintain curcumin concentration in the blood. Besides, the encapsulated curcumin was protected from external conditions; therefore, the curcumin-loaded PECs could directly carry the bioactive curcumin to cancerous lesions in the colon and efficiently destroyed carcinomas, as evidenced by the results of the internalizing experiments (Figure 6). The cell viability results (Figure 7) indicated that the curcumin-loaded PECs exhibited more significant cytotoxicity to HCT116 cells than free curcumin. The results of cell cycle distribution test (Figure 8) further explained the reason. The cell cycle test results showed that curcumin-treated with mimetic GI tract conditions no longer caused any cell cycle arrest, whereas curcumin-loaded PECs triggered the G2/M phase arrest in HCT116 cells, supporting the results of the studies conducted by A. Jaiswal et al. [38] and J. Lu et al. [39]. Our results have greatly supported that our PECs efficiently preserved the therapeutic effect of curcumin as a payload until it reached carcinomas in the colon.

Curcumin-loaded PECs possessed sustained-release properties and could transport active curcumin into tumorous lesions in the colon after treatment with mimetic GI tract conditions. The results of cell viability tests also confirmed the anti-cancer efficacy of the curcumin-loaded PECs. However, the mimetic GI tract conditions in our study did not involve enzymatic effects, and those conditions were also far from simulating both fasted state gastric conditions (FaSSGF) and fasted state conditions in the small intestine (FaSSIF), as recorded in the pharmacopeia [40]. Future application of curcumin-loaded PECs as a new formulation of oral curcumin solution is still being investigated further but worth expecting.

## 4. Materials and Methods

### 4.1. Materials 

Type B gelatin (molecular weight: 5 k; bloom number: 225), sodium phosphotungstate (PTA) for TEM negative staining and (3-(4,5-dimethylthiazol-2-yl)-2,5-diphenyltetrazolium bromide (MTT) powder for cell viability assay were all purchased from Sigma-Aldrich Co., Ltd. (St. Louis, MO, USA). Pectin (methylation ratio: 3%–7%) was obtained from Wako Pure Chemical Industries, Ltd. (Osaka, Japan). Curcumin was acquired from Merck Life Science (Hohenbrunn, Germany). Dialysis bags (M.W.C.O.: 6–8 k) for drug-release assessment were also purchased from Merck Life Science. Ethanol was purchased from Taiwan Sugar Corp. (Tainan, Taiwan). Methanol for drug-loading test was obtained from Alps Chem Co., Ltd. (Hsinchu, Taiwan). Reagents for pH value adjustment, hydrochloric acid and sodium hydroxide, were purchased from J. T. Baker Inc. (Center valley, PA, USA) and Uniregion Bio-tech Inc. (New Taipei, Taiwan), respectively. Biological agents, including dimethyl sulfoxide for preparing curcumin solution in cell viability tests and a Muse Cell Cycle Kit was purchased from Merck Millipore (Burlington, MA, USA). Fluorescent reagents, including Cy 5.5-NHS ester, Lysotracker DND-Red, and DAPI-containing mounting medium, were acquired from Lumiprobe (Hunt Valley, MD, USA), Thermo Fisher Scientific (Waltham, MA, USA), and Vector Laboratory (Burlingame, CA, USA), respectively.

### 4.2. Preparation and Characterization of Pectin-Type B Gelatin PECs

Pectin (10 mg) was weighed and dissolved in 50 mL of deionized water. Type B gelatin (50 mg) was also weighed and dissolved in 50 mL of deionized water at 25 °C. After complete dissolution, the pH of the produced type B gelatin was adjusted to 4.0 using 0.1 N HCl. Ethanol (0.1 mL) was mixed with type B gelatin solution under stirring for 30 min. Next, the pectin solution was added into the mixture, blended, and the pH of the mixture was further adjusted to 4.0. Thirty minutes later, PECs were acquired and stored at 4 °C. To optimize the preparation and component of PECs, various volumes of type B gelatin and pectin were added to form the PECs. The type B gelatin/pectin PECs composed of various amounts of pectin and type B gelatin were collected and measured by DLS (Malvern ZS 90, Malvern Instruments, Malvern, UK) for particle size, distribution, and superficial zeta potential (ξ-potential). The hydrodynamic diameter was determined by the Stokes-Einstein equation after DLS measurement. Size distribution was determined through cumulant analysis of the measured intensity autocorrelation function assumed by Gaussian distribution. Zeta potential was obtained through ELS and calculated with Henry’s equation. 

### 4.3. Observation of the Morphology of PECs 

The prepared PEC sample was dropped to carbon-coated copper mesh and excess sample was removed after the PEC sample was attached to the mesh. Sodium phosphotungstate aqueous (PTA) solution 1% (*w*/*v*) was prepared and dropped to the copper grids for negative staining. Excess PTA solution was then eliminated. Next, the copper grids were placed in a vacuum oven. Twenty-four hours later, the dried copper grids were kept from light exposure at room temperature until observation. The morphology of the PECs was observed using TEM (HT7700, Hitachi, Japan) with an accelerated voltage of 120 V under high contrast mode (HC mode). 

Besides, the morphology of PECs was also observed using a thermal field emission scanning electron microscope (Thermal FE-SEM) (JEOL JSM-7800F, JEOL Ltd., Tokyo, Japan). The PEC solutions were first prepared and freeze-dried using a vacuum freeze drier (Uniss MF-280, Uniss Company, New Taipei City, Taiwan). The freeze-dried powder was afterwards coated with platinum and observed by the thermal FE-SEM. 

### 4.4. pH Responsiveness of PECs

PECs (5 mL) were placed into a sample vial and its pH was adjusted to 2.0 using HCl (0.1 N) to mimic the acidic condition in the stomach. Afterward, the PEC solution was incubated at 37 °C for 2 h in a shaker (M.BR-022UP, Taitec, Tokyo, Japan). Next, the pH of the PEC solution was adjusted to 8.0 using NaOH (0.5 N) to simulate the alkaline environment in the small intestine. The adjusted PEC solution was placed at 37 °C for 4 h in a shaker. Particle size and surface zeta potential were measured by DLS at every hour. 

### 4.5. Preparation and Characterization of Curcumin-Loaded Pectin-Type B Gelatin PECs 

Different amounts of curcumin (0.5, 1, 1.5, 2, or 2.5 mg) were precisely weighed and placed independently in a vial. Curcumin was first dissolved in ethanol (0.1 mL). Simultaneously, pectin (10 mg) and type B gelatin (50 mg) were respectively weighed and dissolved in 50 mL deionized water as stock solutions. Type B gelatin solution was treated with 0.1 N of HCl until the pH was 4.0. Curcumin alcoholic solution (0.1 mL) and type B gelatin solution (0.2 mL) were first mixed in a sample vial with stirring for 30 min. Thereafter, pectin solution (0.7 mL) was added to the mixture, which was then blended for 30 min to fabricate curcumin-loaded PECs. Particle size and distribution were determined using DLS.

### 4.6. Drug Contents and Loading Efficiency Determination

Curcumin-loaded PECs were prepared as abovementioned. The prepared curcumin-loaded PECs were freeze-dried using a vacuum freeze drier (Uniss MF-280). The dried sample (0.5 mg) was then weighed and dissolved in 1 mL of methanol for curcumin extraction. The extracted curcumin was quantified by an ELISA reader (BioTek Synergy HT, BioTek Instruments, Inc., Winooski, VT, USA) at 423 nm as the detected wavelength. 

The drug content of the curcumin-loaded PCs was calculated according to the formula (1):

(1) $$\mathrm{Drug}\;\mathrm{contents}\;\left(\%\right)=\frac{weight\;of\;encapsulated\;curcumin\;\left(mg\right)}{total\;weight\;of\;curcumin-loaded\;PECs\;\left(mg\right)}\times 100\%$$

Besides, drug loading efficiency was also determined and calculated according to the formula (2):

(2) $$\mathrm{Loading}\;\mathrm{efficiency}\;\left(\%\right)=\frac{encapsulated\;curcumin\;weight\;\left(mg\right)}{total\;curcumin\;input\;weight\;\left(mg\right)}\times 100\%\;$$

After calculation, the optimization was analyzed with analysis of variance (ANOVA).

### 4.7. Determination of Drug-Releasing Profiles

Curcumin-loaded PECs (1 mL) were placed into dialysis bags, which were then immersed into 2.5 mL of methanol/deionized water solution (1:1, *v*/*v*) in a sample vial. The pH of the methanol/deionized water solutions in a sample vial was first adjusted to 2.0, and then the sample vial was incubated at 37 °C for 2 h. Afterwards, the pH value of the methanol/deionized water solution in the sample vial was adjusted to 8.0, and the sample vial was further incubated at 37 °C for 4 h. At each hour, 0.1 mL of methanol/deionized water solution was collected and absorbance wavelength at 423 nm was detected to quantify the release of curcumin.

### 4.8. CLSM Observation

HCT116 cells (1 × 10^5^ cells/mL) were seeded on a slide and incubated at 37 °C with 5% CO_2_ supply for 12 h. After the cells were attached, they were co-cultured with free curcumin- or Cy5.5-labeled curcumin-loaded PECs, which were pretreated with mimetic GI tract conditions for 12 h at 37 °C with 5% CO_2_ supply. Excess curcumin or curcumin-loaded PECs were thereafter removed from the cells, which were then washed twice with PBS. The cells were further stained with 1 µM of Lysotracker DND-Red at 37 °C with 5% CO_2_ for 1 h. Next, the lysotracker DND-Red was removed, and the cells were again washed twice with PBS and fixed with 4% paraformaldehyde for 20 min. After fixation, the cells were washed twice with PBS and the cells and their nuclei were stained and mounted with DAPI-containing mounting medium. Fluorescence was observed and detected using a confocal laser scanning microscope (Leica SP8, Leica Microsystem, Wetzlar, Germany). The fluorescence of Cy 5.5 was detected at excitation and emission wavelengths of 633 and 650 nm, respectively. The fluorescence of Lysotracker DND-Red for locating endosome/lysosomes was detected using excitation and emission wavelengths of 577 and 590 nm, respectively. The fluorescence of curcumin was observed at excitation and emission wavelengths of 488 and 520 nm, respectively. DAPI fluorescence was detected using the preset wavelength of the CLSM equipment.

### 4.9. Cell Viability Determination

Human colon cancer cells HCT116 (7 × 10^3^ cells/mL) were seeded on a 96-well plate at 37 °C with 5% CO_2_ supply for 12 h to allow cell attachment. Simultaneously, curcumin-loaded PECs were adjusted with the cell culture medium to the predetermined concentrations at 45 to 1.41 µM. Curcumin solutions at the same concentration series were also prepared with 1% of DMSO and cell culture medium. Afterwards, the pH of both curcumin and curcumin-loaded PECs was adjusted to 2.0 for 2 h, then the pH of the PECs was adjusted to 8.0 for 4 h. The pH of the PECs was re-adjusted to 7.4, and then HCT116 cells were treated with the PECs. The HCT116 cells were incubated at 37 °C with 5% CO_2_. Twelve hours later, the PECs and cell culture medium were removed. MTT assay was utilized to evaluate cell viability.

In addition, the human normal fibroblast cells Detroit 551 (7 × 10^3^ cells/mL) were also seeded on a 96-well plate and cultured at 37 °C with 5% CO_2_ for 12 h. Bare PECs at concentrations of 112.5 to 3.52 µM were prepared with serial dilution. The bare PECs were separated into two groups: one for direct treatment with Detroit 551 cells and the other for mimetic GI tract treatment, as described above. Next, the latter bare PECs were transferred to pH 7.4 condition, and the cells were cultured with the bare PECs for 12 h. MTT assay was then applied to determine cell viability.

### 4.10. Cell Cycle Analysis

HCT116 cells (3 × 10^6^ cells/mL) were seeded on a 6-well plate and cultured for 12 h at 37 °C with 5% CO_2_ supply. After the cells were attached, they were exposed to curcumin or curcumin-loaded PECs pretreated in mimetic GI tract conditions (pH 2.0 for 2 h, pH 8.0 for 4 h, and back to pH 7.0), as described above, and then the cells were incubated at 37 °C with 5% CO_2_ supply for 12 h. Afterward, the cells were collected, washed with PBS, and fixed with 70% of ethanol at −20 °C for 6 h. Fixed cells were re-collected using centrifugation and suspended in PBS. The cells were further stained with a flow cell cycle kit in the dark for 30 min. Cell cycles were determined using flow cytometry (BD FACSCanto, BD Biosciences, Franklin Lakes, NJ, USA) and analyzed with the software ModFit LT 3.0 (BD Biosciences, Franklin Lakes, NJ, USA).

### 4.11. Statistical Analysis

All experiments were repeated at least thrice and the results were averaged and presented as mean ± standard deviation (S.D.). Most results have been statistically compared and analyzed using Student’s *t*-test (Microsoft Excel 2000). Differences were recognized as statistically significant when the *p* values were less than 0.05, and significant differences are shown in star marks (* *p* < 0.05; ** *p* < 0.01, and *** *p* < 0.001). Besides that, of all results, only the optimization of drug contents and loading efficiency were analyzed with analysis of variance (ANOVA) using OriginPro 8 (OriginLab, Northampton, MA, USA).

## 5. Conclusions

In this study, a novel pectin-type B gelatin PEC was fabricated for curcumin delivery and its optimum manufacturing processes, including component ratios and curcumin-loading process, were also developed. The produced PECs could increase their particle size at pH 2.0 for the neutral charges of pectin. At pH 8.0, type B gelatin would be electrically neutralized, driving the gradual swelling of the PECs. The curcumin-loaded PECs could release their payloads at both pH 2.0 and 8.0, which potentiate exploration of their oral formulation. Besides, the PECs could also preserve the bioactivity of curcumin and be internalized into colon cancer cells to allow intracellular drug-releasing behaviors. In addition, the curcumin-loaded PECs induced cell cycle arrest and excellent cytotoxicity toward the human colon cancer cells HCT116 even after treatment under mimetic GI tract conditions, whereas bare PECs by themselves showed very low toxicity to normal cells. The pectin-type B gelatin PEC that possessed high safety and outstanding anti-cancer effects is a promising oral formulation for curcumin delivery.

## Figures and Tables

**Table 1:** The particle size and distribution of the PECs containing various ratios of constituents (to a final concentration of 1 mL).

Code	Components		Particle Size (nm)	PDI
	Pectin	Type B Gelatin		
	(mol %)	(mol %)		
P1G9	10	90	2745.0 ± 12.8	0.15 ± 0.08
P2G8	20	80	1502.8 ± 243.0	0.40 ± 0.10
P3G7	30	70	364.7 ± 6.4	0.13 ± 0.09
P4G6	40	60	264.0 ± 3.1	0.23± 0.02
P5G5	50	50	311.0 ± 57.8	0.18 ± 0.02
P6G4	60	40	243.1 ± 18.8	0.23 ± 0.10
P7G3	70	30	259.3 ± 21.6	0.17 ± 0.12
P8G2	80	20	276.5 ± 28.0	0.21 ± 0.07
P9G1	90	10	278.9 ± 57.1	0.25 ± 0.08

## Supplementary Materials

Supplementary materials can be found at http://www.mdpi.com/1422-0067/19/11/3625/s1.

## Abbreviations

PEC	polyelectrolyte complex
GI tract	gastrointestinal tract
pI	isoelectric point
DLS	dynamic laser scattering
PDI	polydispersity index
TEM	transmission electron microscopy
ELISA	enzyme-linked immunosorbent assay
M.W.C.O.	molecular weight cutoff
MTT	(3-(4,5-dimethylthiazol-2-yl)-2,5-diphenyltetrazolium bromide)
CLSM	confocal laser scanning microscope
DAPI	4′,6-diamidino-2-phenylindole
