# Supplementary material for: Development of Pectin-Type B Gelatin Polyelectrolyte Complex for Curcumin Delivery in Anticancer Therapy
Source: Int J Mol Sci. 2018 Nov 17;19(11):3625. doi: 10.3390/ijms19113625 (PMC6274754; doi:10.3390/ijms19113625)
Supplement: Supplementary file 1 [file ijms-19-03625-s001.pdf]

Supporting information

Development of Pectin-Type B Gelatin Polyelectrolyte Complex for Curcumin Delivery in Anticancer Therapy

Fu-Ying Shih [1,#], Ih-Jen Su [2,3,4,#], Li-Lun Chu [5], Xiaojie Lin [6], Sheng-Chu Kuo [7,8], Yu-Chi Hou[5] and Yi-Ting Chiang [5,*]

[1] Ph.D. Program for Biotech Pharmaceutical Industry, College of Pharmacy, China Medical University, Taichung 404, Taiwan; u106308001@cmu.edu.tw

[2] Department of Biotechnology, Southern Taiwan University of Science and Technology, Tainan 710, Taiwan.; suihjen0704@stust.edu.tw

[3] Department of Pathology, National Cheng Kung University Hospital, Tainan 704, Taiwan; suihjen0704@stust.edu.tw

[4] National Institute of Infectious Diseases and Vaccinology, National Health Research Institutes, Tainan 704, Taiwan; suihjen0704@stust.edu.tw

[5] School of Pharmacy, China Medical University, Taichung 404, Taiwan; u103003505@cmu.edu.tw, houyc@mail.cmu.edu.tw & ytchiang@mail.cmu.edu.tw

[6] Department of Chemical Engineering, University of Washington, Seattle, Washington 98195, U.S.A.; xjlin@uw.edu

[7] Chinese Medicine Research Center, China Medical University, Taichung 404, Taiwan; sckuo@mail.cmu.edu.tw

[8] Research Center for Chinese Herbal Medicine, China Medical University, Taichung 404, Taiwan; sckuo@mail.cmu.edu.tw

[#] These authors contributed equally to this manuscript

* Correspondence: ytchiang@mail.cmu.edu.tw; Tel.: +886-4-2205-3366 ext. 5611

Supporting information-1

Figure SI-1. Scanning electron microscopic images (SEM images) of P4G6 (a) and P6G4 (b) polyelectrolyte complexes (PECs).

(a)

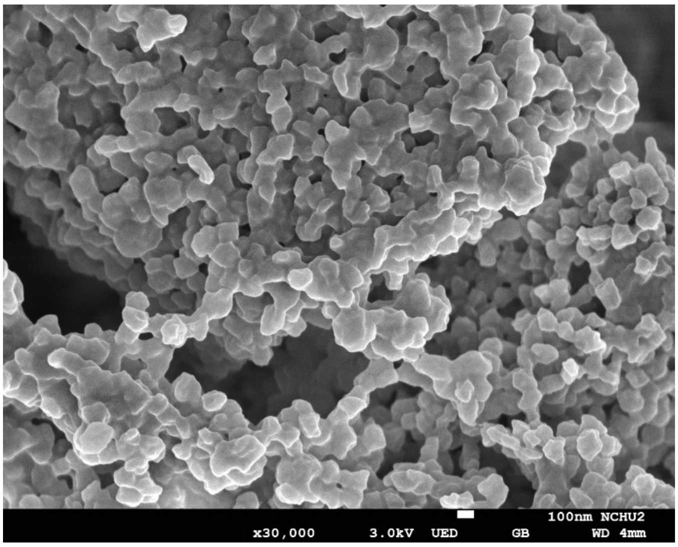

(b)

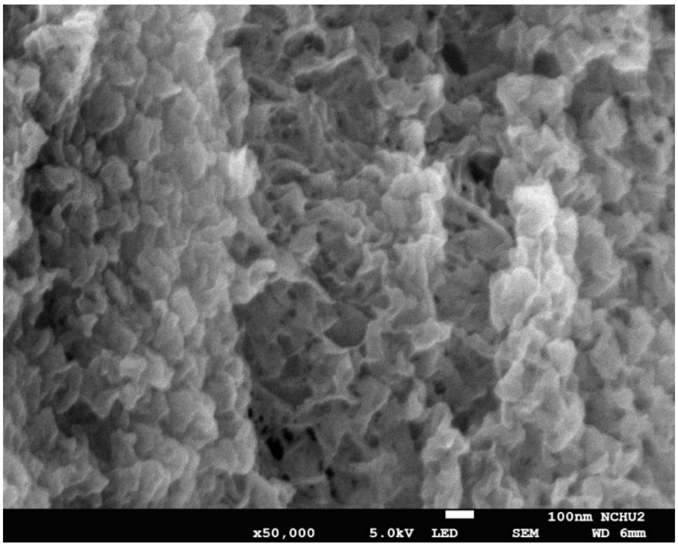

Figure SI-1. SEM images of P4G6 (a) and P6G4 (b) PECs. The PEC solutions were prepared and freeze-dried. The freeze-dried powder were suppter coated with platinum and the morphology of P4G6 (a) and P6G4 (b) PECs were analyzed using a scanning electron microscopy (SEM).

Supporting information-2

Figure SI-2. Cell viability of bare PECs toward human fibroblast cells Detroit 551.

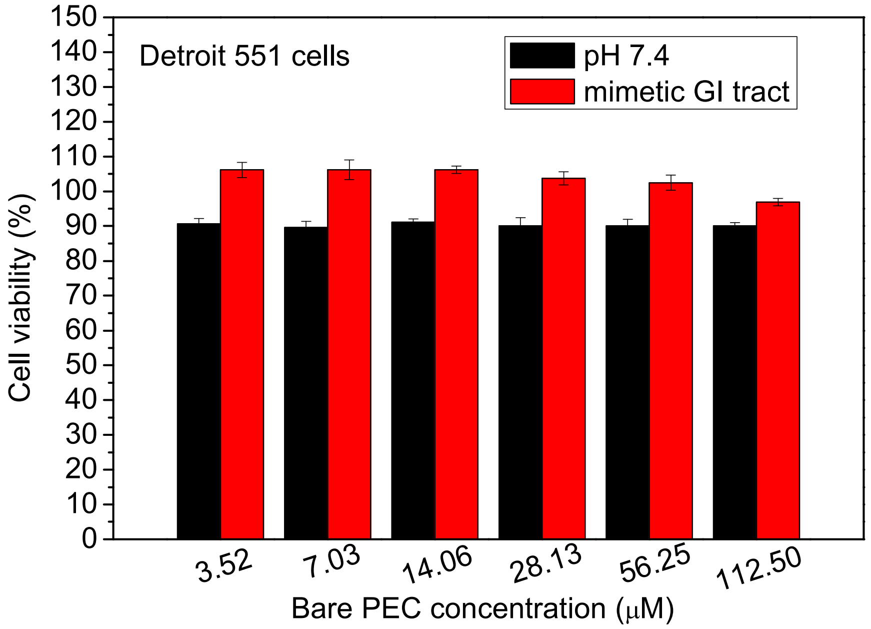

Figure SI-2. Cell viability of bare PECs toward human normal fibroblast cells Detroit 551. Human normal fibroblast cells Detroit 551 cells were seeded on a 96-well plate and cultured for overnight. Bare PECs were incubated at pH 2.0 for 2 h and transferred into pH 8.0 conditions for 4 h to mimetic GI tract. Afterwards, the bare PECs were treated with cells for 12 h, simultaneously the bare PECs without acid or alkaline treatment were also incubated with Detroit cells at pH 7.4. The cell viability was evaluated using MTT assay.
